# Complete Genome Sequence of a Metronidazole-Resistant Helicobacter pylori Strain

**DOI:** 10.1128/genomeA.00051-15

**Published:** 2015-03-12

**Authors:** Tran Thanh Binh, Rumiko Suzuki, Dong Hyeon Kwon, Yoshio Yamaoka

**Affiliations:** a Department of Environmental and Preventive Medicine, Oita University Faculty of Medicine, Oita, Japan; b Biology Department, Long Island University, Brooklyn, New York, USA; c Department of Medicine-Gastroenterology, Baylor College of Medicine and Michael E. DeBakey Veterans Affairs Medical Center, Houston, Texas, USA

## Abstract

We report here the complete genome sequence of a metronidazole-resistant Helicobacter pylori strain (MET^r^). The MET^r^ strain was obtained under exposure of H. pylori 26695 on agar plates with low metronidazole concentrations. The genome data provide insight into the genomic changes of H. pylori under selection by metronidazole *in vitro*.

## GENOME ANNOUNCEMENT

Helicobacter pylori is a spiral Gram-negative bacterium that infects more than half of the world’s population and is a major cause of chronic gastritis, peptic ulcer diseases, gastric cancer, and mucosa-associated lymphoid tissue lymphoma (MALT) (1, 2). The eradication of H. pylori not only improves peptic ulcer healing but also prevents its recurrence and reduces the risk of developing gastric cancer (3–6). Metronidazole has been widely used in combination with therapies, such as metronidazole-based triple therapy, concomitant therapy, and bismuth-containing quadruple therapy, to eradicate this bacterium (6–8). Although treatment success depends on several factors, such as smoking status and patient compliance, antibiotic resistance is the major cause (9–11). However, along with clarithromycin resistance, resistance to metronidazole has arisen independently and is becoming increasingly common (12, 13).

In the present report, we announce the genome sequence of a metronidazole (MET)-resistant (MET^r^) H. pylori strain obtained *in vitro*. The genome of the strain provides insight into the genomic changes of H. pylori under MET selection.

The MET^r^ strain was obtained under the exposure to a low concentration of MET *in vitro* (up to 16.0 µg/ml MET in an agar plate), and whole-genome sequencing was performed using 90-base paired-end reads on the Illumina HiSeq 2000 genome sequencer (Illumina, Inc., San Diego, CA). The whole-genome sequence of the strain was reconstructed by mapping the short-read sequences on the genome of H. pylori 26695 (GenBank accession no. NC_000915) using CLC Genomics Workbench version 4.0 (CLC bio, Aarhus, Denmark). The length of 26695-1MET genome is 1,667,302 bp, with a coverage depth of 680×. The G+C content of this strain is 38.87%.

Three mutations in 3 genes were found in the H. pylori MET^r^ strain, including the mutations in the *rdxA* (*hp0954*) and *frxA* (*hp0642*) genes (14). The new mutation (G37T) was found in the gene *rpsU* (*hp0562*).

### Nucleotide sequence accession number.

The genome sequence of the metronidazole-resistant H. pylori strain (26695-1MET) was deposited at GenBank under the accession no. CP010436.
